# Estimating the Abundance of an Endangered Arboreal Marsupial Using Camera Traps and an Integrated Species Distribution Model

**DOI:** 10.1002/ece3.72037

**Published:** 2025-09-01

**Authors:** Yiyin Chang, Chieh Lin, Conrad J. Hoskin

**Affiliations:** ^1^ College of Science and Engineering James Cook University Townsville Australia

**Keywords:** abundance estimates, camera trapping survey, conservation, integrated species distribution model, mahogany glider, *Petaurus gracilis*, threatened species

## Abstract

Estimates of abundance are fundamental for the management and conservation of threatened species. The Mahogany Glider ( *Petaurus gracilis* ) is an Endangered marsupial endemic to the Wet Tropics of northeastern Australia. Despite its status, there is no reliable estimate of abundance. In this study, we conducted camera trapping surveys for the species and employed a Bayesian integrated species distribution model to derive abundance estimates. Presence–absence data from camera trapping surveys and presence‐only data from historical sighting records were included in the integrated species distribution model. The model estimated median abundance at 6036, 4834 and 2820 individuals for home range estimates of 9, 16 and 25 ha, respectively. We suggest using the more conservative abundance estimate of about 2800 individuals, based on the 25 ha home range, because it likely best summarizes density across the distribution. Using simulated data, we tested the effects of camera placement and subsampling, demonstrating that clustered camera arrangements and subsampling from aggregation did not significantly affect model outcomes, with predictions primarily dependent on home range estimates. Our survey results suggest considerable spatial variation in glider density across its range. The abundance estimates provide a baseline for future conservation initiatives and highlight the importance of ongoing monitoring and the application of advanced modeling techniques to inform species management.

## Graphical abstract

We estimated the total abundance of the Endangered Mahogany Glider ( *Petaurus gracilis* ) using camera trapping and a Bayesian integrated species distribution model. Our results suggest a median abundance of 2820 to 6036 individuals, with 2820 being the most conservative estimate. These findings provide a baseline for future monitoring and highlight the importance of advanced modeling techniques in species conservation.

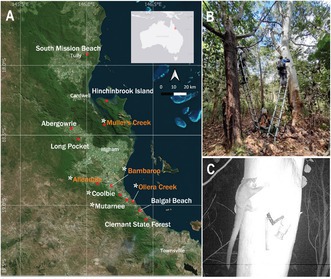

## 1 Introduction

Estimating species abundance presents significant challenges. Because it is impractical to count every individual, abundance estimates must be derived from sampling, which requires the use of statistical models (MacKenzie and Nichols 2004; Bonar et al. 2011; Bailey, Mackenzie, and Nichols 2014). Several widely used approaches exist, each with different data requirements and assumptions. Capture–recapture methods estimate detection probability and abundance by repeatedly surveying marked individuals over time (e.g., White and Burnham 1999). Random Encounter Models treat animals as randomly moving particles and estimate density within a defined area, but they require cameras to be unbaited and randomly placed (Rowcliffe et al. 2008). Site‐structured models, such as the Royle–Nichols model (Royle and Nichols 2003; Royle et al. 2005) and N‐mixture models (Royle 2004), do not require animals to be individually identified or cameras to be randomly placed. These models rely on repeated presence–absence or count data to account for imperfect detection. Distance sampling is another well‐established method that estimates density based on the measured distance to detected individuals (e.g., Buckland et al. 2015). These models rely on assumptions of a closed population during the survey period, independent detections and unbiased detection probabilities (Gilbert et al. 2021), which may not hold in field conditions, particularly for low‐density or cryptic species.

Biases and imperfections in estimating abundance are often amplified for rare species, and the challenges vary depending on the nature of rarity (e.g., clumped vs. dispersed; broad vs. narrow distribution) (Jeliazkov et al. 2022). Rare species typically have low detection rates, which increases the risk of false negatives (instances where animals are present but undetected) leading to underestimation of abundance and potentially misleading inferences about habitat associations (Gu and Swihart 2004; Cunningham and Lindenmayer 2005). High frequencies of non‐detection can also result in inflated zeros in the data, which, if not appropriately modeled, may bias parameter estimates or reduce model fit (Welsh et al. 1996; Dénes et al. 2015). Furthermore, small sample sizes, such as low numbers of detections, reduce statistical power and inflate the variance of estimates, which can disproportionately bias abundance estimates and reduce confidence in model outputs (Gerrodette 1987; Link and Sauer 1997; Bean et al. 2012). To address these issues, either greater survey effort is required to obtain representative data (e.g., Bonar et al. 2011; Linden et al. 2017; Burns et al. 2019), or more robust modeling approaches must be used—particularly those that can incorporate multiple data sources and account for imperfect detection (Jeliazkov et al. 2022).

One such approach is the integrated species distribution model, which has been developed to overcome many of these limitations by combining data from multiple detection methods. For instance, the integrated species distribution model (hereafter ‘integrated model’) enables the simultaneous use of presence–absence and presence‐only data within a unified statistical framework (Koshkina et al. 2017). By linking occupancy modeling with species distribution modeling, it accounts for imperfect detection while incorporating environmental covariates to estimate abundance across large spatial scales. The occupancy component supports abundance estimation by using repeated detection/non‐detection data, while the integration of presence‐only data helps relax the closure assumption typically required in standard occupancy or abundance models, allowing inference beyond the boundaries of structured survey data (Dorazio 2014; Koshkina et al. 2017).

The flexibility of the integrated model makes it especially valuable for rare species, where detections are sparse and conventional methods such as mark‐recapture or N‐mixture models may not be feasible (Gilbert et al. 2020). It is also well suited for situations with patchy or incomplete survey coverage and baited sampling (Mäkinen et al. 2024). A Bayesian implementation of the model has been developed to improve accuracy and quantify uncertainty in parameter estimates (Fidino 2021). Although the model assumes independence between observations and requires careful selection of spatial covariates, it has been shown to perform well in estimating abundance for elusive and rare species. Successful applications include the Yellow‐bellied Glider ( *Petaurus australis* ) in southeastern Australia (Koshkina et al. 2017), large carnivores in New York State (Twining et al. 2024) and Baird's Tapir ( *Tapirus bairdii* ) in Central America (Schank et al. 2017, 2019).

The Mahogany Glider ( *Petaurus gracilis* ) is an Endangered mammal species restricted to the Wet Tropics of northeast Australia. This medium‐sized, gliding marsupial is confined to lowland sclerophyll forests characterised by eucalyptus and melaleuca trees with a grassy understory and distinct seasonal rainfall (Jackson and Claridge 1999; Chang et al. 2022). The glider's habitat requires diverse tree species to provide year‐round nectar for foraging and suitable tree hollows for nesting (Van Dyck 1993; Jackson 1998). Mahogany Gliders are reported to be socially monogamous with home ranges of 6–10 ha in fragmented habitats and 19–24 ha in continuous habitats (Jackson 2000b). Listed as Endangered under Australia's Environment Protection and Biodiversity Conservation Act 1999 (EPBC Act, Threatened Species Scientific Committee 2023), the species has suffered from extensive deforestation due to cattle grazing, sugarcane farming and forestry activities (Jackson et al. 2011).

Conservation efforts to date have focused on collaborating with landowners to maintain and restore habitat, conducting revegetation and installing glider poles to facilitate road crossings (Asari et al. 2010; Jackson and Diggins 2020). However, fundamental knowledge gaps remain, including rigorous estimation of population densities and size across the distribution. To date, population density has been estimated only once, through a capture‐recapture study conducted around 1998 in a small area in the center of the distribution (Jackson 2000a). That study suggested a density of 0.16 individuals per hectare in fragmented habitat and 0.24 in continuous habitat, which led to an extrapolation of 10,000–14,000 individuals across the range in a review of the species' conservation status (Burbidge et al. 2014). In the same review, a speculation of 1200–2000 individuals, lacking any methodological detail, is also presented (Burbidge et al. 2014). Uncertainty around abundance has complicated efforts to assess population trends and the impacts of significant events, such as Cyclone Yasi in 2011, on the viability of the species (Starbridge 2012; Holloway 2013).

In this study, we used data from camera trapping surveys and historical sighting records in an integrated model to estimate the abundance of the Mahogany Glider across its known range, aiming to provide a more rigorous abundance estimate. We also assess the potential uncertainty of these estimates by testing the effects of camera placement, sample size and varying home range sizes. Our camera trapping survey strategies and abundance estimates will serve as a foundation for long‐term monitoring of this threatened species.

## 2 Methods

### 2.1 Study Sites and Timing of Surveys

**FIGURE 1:**
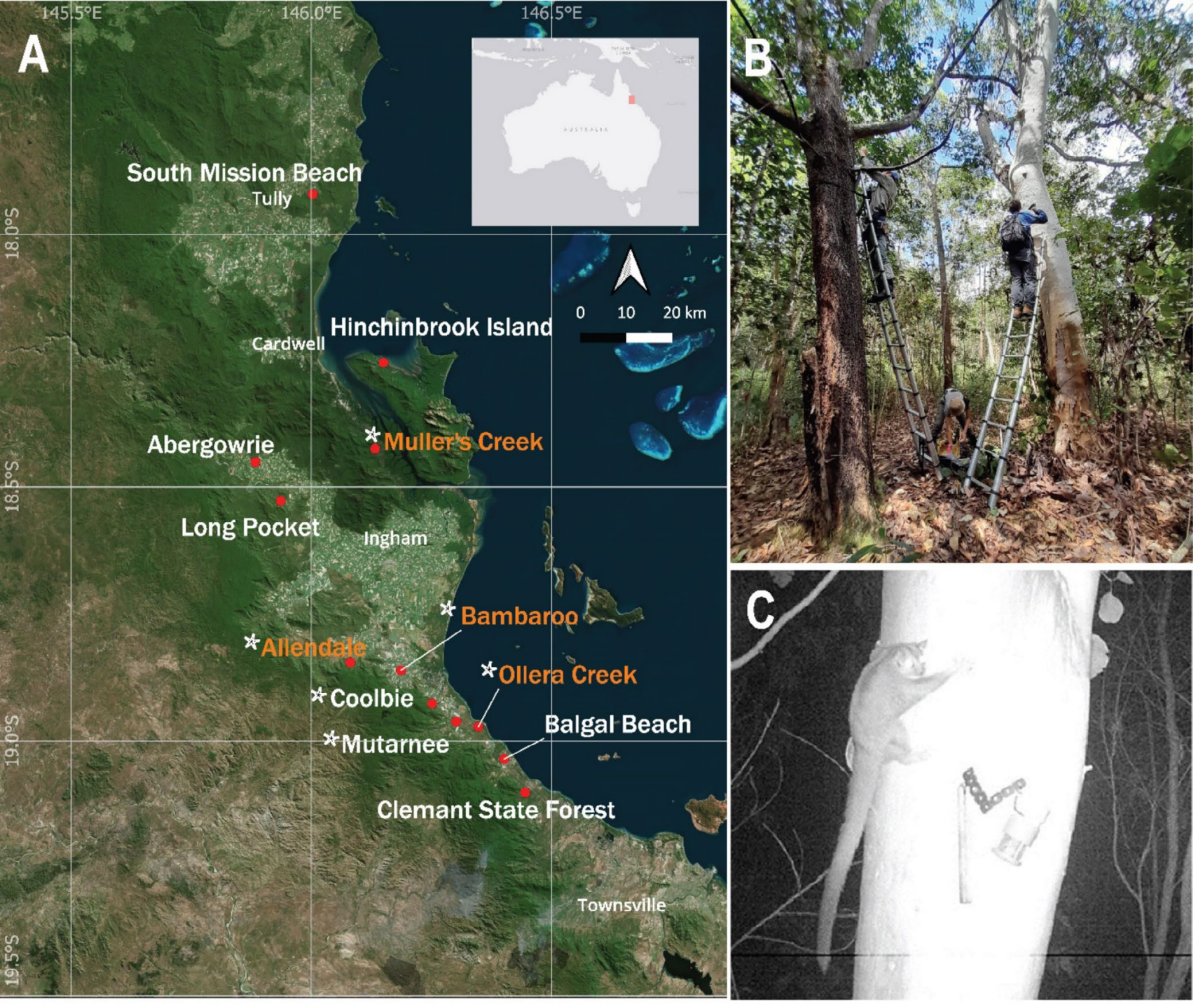
Survey sites and methods. (A) Transect (white) and grid (orange) camera trapping surveys across the Wet Tropics. The asterisks indicate sites where Mahogany Gliders were detected. (B) Installation process of brackets onto the tree pair (one for lure holder, one for camera) for repeat monitoring. Photo credit: Taruna Venkat. (C) A Mahogany Glider captured on a camera trap investigating the lure holder at Muller's Creek. The 21 cm length of wood served as a scale bar.

Between 2020 and 2022, camera trapping surveys were conducted in the lowland woodland of the central and southern Wet Tropics bioregion in north‐east Australia. Two sampling designs were used across different monitoring projects: transects, representing prior‐informed sampling, and grids, representing systematic sampling (Jeliazkov et al. 2022). These data were integrated in the current study to maximize spatial coverage and analytical robustness. Transect surveys were initially conducted to ground‐truth a previously developed species distribution model (Chang et al. 2022), while grid‐based surveys were designed for occupancy and abundance estimation (Figure 1). The details of these two designs are outlined below. Most surveys were carried out during the dry season, primarily from May to November, due to the swampy and inaccessible nature of Mahogany Glider habitat during the wet season (December–April).

### 2.2 Presence‐Absence (PA) Surveys

Both transect and grid designs were used to collect presence‐absence data for the integrated model. The transect sites were monitored using 9 to 24 infrared cameras from 15 to 89 nights, and the grid sites were monitored using a fixed number of 20 cameras from 32 to 45 nights. The cameras were positioned at least 120–150 m apart, depending on the tree availability (Table 1).

**TABLE 1:** Camera trapping survey sites and detection counts of Mahogany Glider (MG).

Site	Region	Survey type	Cameras	Nights	Total trapping effort	MG	MG Naïve rate (%)	Other arboreal species recorded
Abergowrie	West of Ingham	Transect	20	27	540	0	0.00	Krefft's Glider
Balgal	North of TSV	Transect	20	52	1040	0	0.00	Krefft's Glider
Bambaroo	South of Ingham	Transect	24	33–89	1016	19	1.87	NA
Clemant SF	North of TSV	Transect	20	42	840	0	0.00	Krefft's Glider
Coolbie	South of Ingham	Transect	10	53	530	2	0.38	Krefft's Glider, White‐tailed Rat, Feathertail Glider
Hinchinbrook	Cardwell	Transect	20	57	1140	0	0.00	Krefft's Glider
Long Pocket	West of Ingham	Transect	20	15	300	0	0.00	Krefft's Glider
Mutarnee	North of TSV	Transect	20	20	400	2	0.50	Krefft's Glider, Feathertail Glider
South Mission Beach	Tully	Transect	9	58	522	0	0.00	Striped Possum
Allendale	South of Ingham	Grid	20	41–42	832	78	9.38	Krefft's Glider
Bambaroo	South of Ingham	Grid	20	30–31	610	25	4.10	NA
Muller's Creek	Cardwell	Grid	20	44–45	894	22	2.46	Krefft's Glider
Ollera Creek	North of TSV	Grid	20	42	840	3	0.36	Brushtail Possum

*Note:* The location of the sites is shown in Figure 1. Survey design was transect or grid (grey shading). The trapping effort is represented as the product of the total number of nights and cameras at each site. Note that the operational duration of cameras varied within a site, and the variation of each camera was incorporated in the trapping effort calculation and in the model. The Naïve capture rate was calculated by dividing the Mahogany Glider. detections by the total trapping effort.

For transect surveys, camera locations were based on > 50% modelled habitat suitability (Chang et al. 2022), Google Earth satellite imagery, and site accessibility, with exact placement adjusted for habitat conditions and suitable trees. For grid surveys, a grid design was used with 150 m spacing between cameras, created using ArcMap 10.8, and a buffer of 75 m to account for glider home ranges in fragmented habitats (Jackson 2000b). Grid sites had 20 cameras deployed for at least 4 weeks (White 2019; Kays et al. 2020).

At each camera location, a pair of straight‐trunked trees about 2 to 4 m apart was selected. One tree was used to mount the lure holder, while the camera was securely fastened to the other (Figure 1B). To enhance detectability, a mixture of oats, honey and peanut butter was used as lure in custom‐made PVC and metal lure holders. This setup ensures the lure remained functional without being consumed by the gliders or other animals. Additionally, a diluted honey and raspberry cordial solution was sprayed on the lure tree. A 21 cm wooden scale bar (equivalent to the averaged snout‐vent length of Squirrel Gliders) was suspended next to the lure holder for size reference in the images. Swift Enduro professional‐grade motion cameras, equipped with infrared mode and 32 GB SD cards, were used for both survey designs. Infrared cameras were programmed with highest sensitivity to operate from 5 pm to 7 am, during the nocturnal activity period of the glider, capturing sequences of three photos with a 3‐s interval upon detecting animal presence using invisible flash. Cameras were positioned 3 to 4 m above the ground.

In transect surveys, the lure holder was wrapped around the trunk using jute twine and the cameras were securely fastened with tie‐down straps to the tree facing the lure holder. In grid surveys, V‐shaped metal brackets and 10 × 10 cm L‐shaped brackets were installed for the lure holders and cameras, respectively, to standardize the placements for repeat monitoring (Figure 1B,C).

Logistical challenges during arboreal camera trapping included difficulty finding suitably spaced, straight‐trunked trees for consistent placement, overexposure from pale eucalyptus bark in infrared images, and a high rate of false triggers caused by wind‐blown vegetation and rainfall. Surveys were restricted to the dry season (May–November) due to heavy wet‐season rainfall and the inaccessibility of sites, many of which become swampy and impassable during the wet season.

Upon retrieval, photos were viewed manually and animals captured on camera were catalogued. A detection incident was defined as an animal being photographed at least 30 min after the preceding image of that species.

### 2.3 Presence‐Only (PO) Data

We used historical records from the WildNet database and the Mahogany Glider Recovery Team as our presence‐only dataset. Data cleaning involved converting animal counts at each coordinate to presence records, removing entries with incomplete information, and excluding records predating 1990 (due to subsequent landscape changes). This process resulted in a total of 487 valid sighting records in the presence‐only dataset (Chang et al. 2022).

### 2.4 Environmental Covariates

We used the predictors that showed significance in the latest species distribution modelling (Chang et al. 2022) as the environmental covariates in the integrated model (Table S1). These spatial rasters were standardised to the same resolution of 250 m and cropped to the known range of the glider (Blakeney 2015). Soil and vegetation types that were significantly correlated with glider presence were recoded into a binary format (1 for relevant, 0 for others). The preferred soil type was hydrosol, while the preferred vegetation types included *Eucalyptus* woodlands with a tussock grass understory and *Melaleuca* open forests (Table S1). To account for detection bias, we included distance to road, as this factor is known to influence predictions in species distribution modelling (Chang et al. 2022). Missing data representing ocean areas were removed from the environmental covariates. Before model fitting, each variable was scaled and centred (Kruschke 2014).

### 2.5 Integrated Model Description

The integrated species distribution model consists of an inhomogeneous Poisson process that models the latent species abundance across the area of interest using two sub‐models corresponding to two types of monitoring data: (1) a thinned Poisson process that describes the observed presence‐only data, and (2) an occupancy model that fits the presence‐absence data. Both sub‐models are linked to the latent abundance, and the model evaluates the likelihood of all three components simultaneously.

The inhomogeneous Poisson process describes the latent abundance N of the Mahogany Glider in their known extent B as shown in the Equation (1):

(1) $$N\left(B\right)∼\text{Poisson}\;\left(\mu \left(B\right)\right)$$

The mean abundance of Mahogany Glider in the range $\mu \left(B\right)$ is determined by an intensity function λ as shown in Equation (2). In our model, the intensity function depends on an intercept term and nine environmental covariates (Equation 3).

(2) $$\mu \left(B\right)=\int_{B}\lambda \left(s\right)\mathrm{ds}$$

(3) $$\log\left(\lambda \right)=\mathrm{\beta x}{\left(s\right)}^{T}=\beta_{1}\cdot x{\left(s\right)}_{1}+\ldots +\beta_{10}\cdot x{\left(s\right)}_{10}$$

Occupancy models estimate species presence on a spatial grid, and the latent occupancy at gird k ($Z_{k}$) was estimated using the probability of glider presence $\psi_{k}$ through a Bernoulli process (Equation 4) (Fidino 2021). This probability $\psi_{k}$ depends on the latent abundance at the grid k ($N_{k}$) (Equation 6 in Koshkina et al. 2017)

(4) $$Z_{k}∼\text{Bernoulli}\;\left(\psi_{k}\right)$$

Inferring from the latent abundance $N\left(B\right)$, the presence‐only sub‐model composed of a thinned Poisson process describing the imperfectly detected abundance $\pi \left(B\right)$ (Equation 5). The imperfect detection is accounted for by a thinning factor $b\left(s\right)$ in Equation (5), which is composed of an intercept term and an observation bias layer (Equation 10 in Koshkina et al. 2017)

(5) $$\pi \left(B\right)=\int_{B}\lambda \left(s\right)\cdot b\left(s\right)\;\mathrm{ds}$$

The presence‐absence sub‐model is a typical occupancy model which consists of j survey sites and each site surveyed $w_{j}$ days. A successful detection at j site on the w day is a Bernoulli process determined by the latent occupancy Z and observations bias ($p_{j,w}$) as shown in Equation (6).

(6) $$y_{j,w}|Z_{k\left[j\right]}∼\text{Bernoulli}\;\left(p_{j,w}\cdot Z_{\left(k\left[j\right]\right)}\right)$$

We assumed the camera detectability to be consistent across sites, thereby only the intercept term is used in the $p_{j,w}$ function (Equation 4 in Koshkina et al. 2017).

The likelihood functions of Dorazio (2014) and MacKenzie et al. (2002) were used to estimate the coefficients for each environmental covariate in the intensity function of latent abundance and in the thinning factor (biases) for the presence‐only and presence‐absence model, respectively (Koshkina et al. 2017). Assuming the presence‐absence dataset is independent from the presence‐only dataset, the joint likelihood of the integrated model can then be expressed by multiplying the likelihood of the sub‐models (Dorazio 2014; Koshkina et al. 2017).

### 2.6 Testing Model Accuracy

We examined the effects of camera placement, sample size and aggregation methods on the accuracy of the integrated model, based on the simulation framework of Fidino (2021). In the framework, animal occurrence was generated according to environmental covariates within a raster; presence‐only (PO) data were randomly selected across the raster, and 100 cameras were evenly distributed for presence‐absence (PA) data. This simulation process accurately captured the true coefficient values. However, our study differs in two key aspects: (1) our cameras are clustered within 10 sampling sites rather than being evenly distributed, and (2) the number of cameras varies across different resolutions due to subsampling when multiple cameras fall within the same cell. Aggregation, which combines detections from multiple cameras within a cell, reduces both the total number of cameras and detections as the aggregation factor increases (resolution decreases). To ensure the applicability of the model to our data, we conducted simulations to test its ability to cover the true values under the conditions mentioned above. First, we tested the effect of clustered cameras by simulating 25 clusters, each containing four cameras, and compared the modeling results to the evenly distributed 100‐camera setup. Second, we evaluated the impact of sample size changes due to subsampling, without altering individual camera observations. Third, we tested three aggregation methods for each cell using a detectability rate of 0.3: averaging all camera observations (mean), retaining only the camera with the highest detection (max) and summing all observations (sum). The low detectability of 0.3 not only reflects the low detection rate of rare species but also ensures the total observations did not exceed the number of survey nights and hence violate occupancy model assumptions. The assigned coefficients are abundance intercept (Latent Int.), environmental covariate coefficient (Latent Slope), SDM intercept (PO Int.), observation bias (PO slope) and occupancy intercept (PA Int.). The derived values from the assigned coefficients are occupied cells that subsequently estimate the Abundance (Figures S2–S4). The effects were examined by comparing the width of credible intervals of the estimated coefficients and whether they overlap the known true values.

### 2.7 Model Configuration

We fitted the models based on the script of Fidino (2021) (GitHub repository: https://github.com/mfidino/integrated‐occupancy‐model) using JAGS 4.3.1 (Plummer 2003) through the runjags package 2.2.1–7 (Denwood 2016) in R 4.1.2 (R Development Core Team 2022).

Since the environmental variables were represented as spatial rasters, a resolution had to be defined. We aggregated pixels using factors of 3–5, following the methodology of Fidino (2021), to capture the home range estimates for the Mahogany Glider (9–25 ha) (Jackson 2000b). The aggregation factor represents the side length of a grid cell, resulting in three grid sizes: 9 ha (3 × 3), 16 ha (4 × 4) and 25 ha (5 × 5) (Schank et al. 2019; personal communication with Mason Fidino). This range of grid sizes captures the variability in home range estimates (9–25 ha) and was used to estimate total abundance under different home range scenarios (Jackson 2000b). When multiple cameras were present within a single aggregated grid, only the camera with the most detections was retained, based on the optimal method identified in the simulation tests.

The process involved 4 chains, with a 1000‐step adaptation phase, followed by a 10,000‐step burn‐in, and 25,000 steps with a thinning factor of 5 to avoid auto‐correlation. We subsequently sampled the posterior 5000 times on each chain, which resulted in a total of 20,000 posterior samples. To ensure model convergence, we visually inspected the trace plots for each variable and verified Gelman‐Rubin diagnostics were less than 1.05 (Brooks and Gelman 1998). The Gelman‐Rubin statistic, also known as the R‐hat statistic, compares the variability within individual chains to the variability between different chains. We then determined the evidence of an effect by calculating 95% credible intervals (CIs) for each variable and assessed whether they overlapped zero.

To capture the range of abundance estimates, we drew 1000 sets of model coefficients from the posterior distribution and generated 1000 corresponding abundance predictions. For each prediction, total abundance was calculated by summing the values across all pixels. Finally, we assessed the most likely abundance estimates and their uncertainty by examining the quantiles of the resulting abundance distribution (posterior prediction).

### 2.8 Abundance Estimates Comparison

The abundance estimates from the integrated model were compared to two previous estimates reported in Burbidge et al. (2014) and two extrapolated estimates based on data from Jackson (2000b) and Jackson et al. (2019). The estimates from Burbidge et al. (2014) included an extrapolated abundance of 10,000–14,000 individuals (based on a density estimate of 0.16 individuals per hectare in fragmented habitat and 0.24 in continuous habitat; Jackson 2000a) and a hypothesized abundance of 1500–2000 individuals (based on unpublished genetic data and personal communication with Mark Parsons).

**FIGURE 2:**
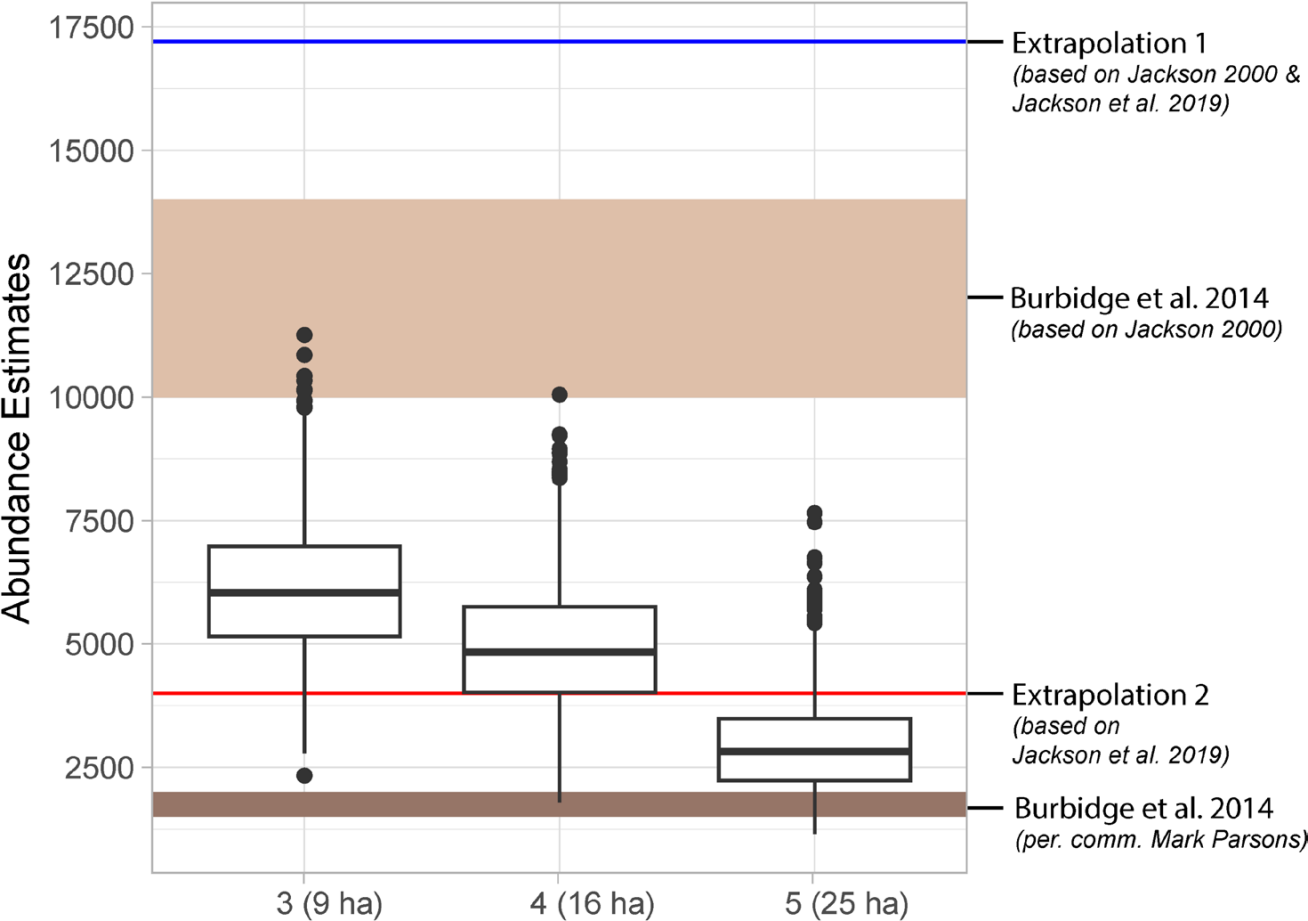
Posterior predictions of the total abundance of Mahogany Gliders across their known range at home range sizes of 9, 16 and 25 ha (aggregation factors 3–5). The Boxplots represent estimates from the 1000 predictions from the integrated species distribution model. The brown ribbons indicate estimates mentioned by Burbidge et al. (2014). The blue and red lines represent extrapolations based on density estimates and habitat remnant assessments from Jackson (2000a, 2000b) and Jackson et al. (2019) (see Section 2).

We calculated two conservative abundance estimates based on table 1 in Jackson et al. (2019), which lists the areas of primary and secondary subpopulations, along with associated habitat fragments, within the distribution of the Mahogany Glider. For the first estimate, we extrapolated abundance by multiplying the total area of the five primary subpopulations (each considered viable with > 800 individuals) by the estimated density for fragmented habitats (0.16 individuals/ha). This resulted in an estimated total abundance of 107,483 × 0.16 = 17,197 individuals (Extrapolation 1 in Figure 2). We did not include secondary subpopulations in this estimate because Jackson et al. (2019) indicated that these populations are only viable with the establishment of corridors. In the second extrapolation, we assumed each of the five primary subpopulations supported only the minimum viable abundance of 800 individuals, resulting in a total estimated population of 4000 individuals (Extrapolation 2 in Figure 2).

## 3 Results

### 3.1 Camera Trapping Surveys

The surveys conducted within the known range of the Mahogany Glider generally recorded low detection rates, except for three sites that exhibited relatively high detection rates: Allendale, Bambaroo and Muller's Creek (Table 1). Allendale is situated south of Ingham in fairly continuous habitat; Bambaroo is a small and isolated fragment of primary forest near Allendale, and Muller's Creek is located further north, between Ingham and Cardwell (Figure 1). Coolbie, Mutarnee and Ollera Creek, positioned at the southern end of their range (Figure 1), had low detection rates, with only one to three observations each (Table 1). Surveys at Abergowrie and Long Pocket, both situated in high‐probability habitat areas to the west of Ingham (Figure 1), did not yield any Mahogany Gliders (Table 1).

Four of the survey sites were outside of (but close to) the known distribution of the Mahogany Glider, namely Hinchinbrook Island, Clemant State Forest, Balgal Beach and South Mission Beach (Figure 1A). Our surveys failed to detect any Mahogany Gliders in these areas. It is worth noting that the surveys on Hinchinbrook Island could only be conducted in moderate suitability habitat because high suitability habitat was not accessible (Chang et al. 2022).

In addition to the target species, other arboreal mammals detected during the surveys included the Common Brushtail Possum ( *Trichosurus vulpecula* ), White‐tailed Rat ( *Uromys caudimaculatus* ), Krefft's Glider (*Petaurus notatus*), Feathertail Glider ( *Acrobates pygmaeus* ), Striped Possum ( *Dactylopsila trivirgata* ) and Fawn‐footed Melomys ( *Melomys cervinipes* ).

### 3.2 Testing Model Accuracy

In the parameter testing, we used simulated data to assess the effects of camera placement (clustered vs. evenly distributed), sample size and aggregation methods (Figures S2–S4).

At finer spatial resolutions (aggregation factors 5 and 10), the model exhibited increased uncertainty in occupied cells. In contrast, at coarser resolutions (aggregation factors 15 and 20), the model tended to underestimate the number of occupied cells (Figures S2 and S3) and may fail to capture the true values (Figure S1). Clustered camera placement reduced the stability and accuracy of estimated coefficients and occupied cells at higher aggregation factors (15 and 20), although most estimated ranges still captured the true values (Figure S2). In contrast, sample size reduction through subsampling had minimal impact on model performance (Figure S3).

As the aggregation method influences the occupancy sub‐model and its associated abundance intercept, we assessed the performance of three aggregation approaches (max, mean and sum) using simulated data. The mean method performed poorly, showing high variability and instability in estimates of the latent abundance intercept, occupancy intercept (PA Int., which reflects detectability), the number of occupied cells, and total abundance (Figure S4). While the max and sum methods performed similarly, the sum method may inflate detection counts, particularly under high detectability, resulting in values that exceed the number of survey nights and violate occupancy model assumptions. Therefore, we selected the max method (which uses the highest detection value from cameras within each grid cell) for our model (Figure S4). This approach is also biologically meaningful, as the aggregation factor defines the pixel size used to approximate home range, and the maximum detection value better reflects the number of resident individuals within that area.

### 3.3 Abundance Estimates Using the Integrated Model

As observed in the simulation tests, abundance estimates were scale‐dependent, with the use of smaller home range estimates producing higher abundance estimates (Figure 2). Therefore, we estimated the total abundance using three home range scenarios (grid sizes) that cover the estimated home range of Mahogany Glider (Jackson 2000b). For the smallest estimated home range of 9 ha (aggregation factor = 3), the quantile ranged from 5155 to 6977 individuals, with a median of 6036. For the middle home range estimate of 16 ha (aggregation factor = 4), the quantile ranged from 4018 to 5755, with a median of 4834. For the largest home range of 25 ha (aggregation factor = 5), the quantile ranged from 2236 to 3487, with a median of 2820 (Figure 2, Table S2).

The overall estimated detectability, based on the occupancy sub‐model of the integrated model, ranged from 4.5% to 8.7% across aggregation factors 3 to 5. These values reflect the low detection rates observed during field surveys. The coefficients representing species–environment relationships, which quantify the effect of environmental covariates on predicted species abundance and occupancy, remained relatively consistent across the aggregation factors. Most covariates were effective predictors, as indicated by their credible intervals not overlapping zero, except for the minimum temperature of the month, which showed no significant effect (Figure S1). Among the coefficients, the occupancy intercept exhibited greater variability compared to the abundance and SDM intercepts. The most important environmental covariates in the models were distance to roads (−), elevation (−), preferred vegetation type (Eucalyptus open woodlands) (+) and temperature seasonality (+) (Table S2). Distance to waterways (+) was also significant across all three models, but to a lesser degree (Table S2). Soil type, precipitation seasonality and fire frequency were positively correlated with predicted densities, but their magnitude of effect varied between models (Table S2).

## 4 Discussion

Here we used presence‐absence data from camera trapping surveys and presence‐only data from historical sighting records in an integrated species distribution model, with the aim of providing the first detailed abundance estimate of Mahogany Glider. The integrated model estimated a median Mahogany Glider abundance of 6036 individuals (based on a 9 ha home range), 4834 (16 ha) and 2820 (25 ha). These estimates fall between previous coarse population bounds of 1200–2000 and 10,000–14,000 individuals (Burbidge et al. 2014; Jackson et al. 2019). We consider the estimate based on a 25 ha home range (approximately 2800 individuals) to be the most likely total abundance of Mahogany Glider because most of the total habitat area is concentrated in a small number of large and medium‐sized patches, and the thousands of tiny, fragmented patches are unlikely to support viable populations (Jackson 2000b; Chang et al. 2022). We highlight two key advantages of applying the integrated model for abundance estimates. First, it accommodates low detectability and reduces uncertainty by combining sighting records with repeated presence‐absence data, outperforming expert opinions and standalone abundance‐occupancy models (Koshkina et al. 2017). Second, the integrated model enables estimation across entire distributions rather than specific study areas, with quantifiable confidence intervals (e.g., Schank et al. 2017, 2019; Twining et al. 2024).

### 4.1 The Effect of Aggregation Factors in Integrated Model Estimates

The integrated model is sensitive to the aggregation factor, which in our model represents the species' estimated home range. As the aggregation factor decreases (i.e., resolution increases), abundance estimates rise. This scale dependency is inherent when working with gridded spatial data, when accounting for false positives, and in the absence of individual identification—a common challenge in most camera trapping surveys (Steenweg et al. 2018; Nakashima 2020). This scale dependency was not addressed in the original model (Koshkina et al. 2017) but was later confirmed in a study on Baird's Tapir ( *Tapirus bairdii* ) using the same model (Schank et al. 2019). Our analysis shows the same pattern of scale dependency. Defining the aggregation factor based on home ranges is biologically meaningful, but this process can be complex when the home range varies across the range or is difficult to determine (e.g., Schank et al. 2019; Fidino 2021). Incorporating variation in home range estimates, and using averaged home range estimates, are therefore recommended approaches (Schank et al. 2019).

### 4.2 Covariates in the Integrated Model

Most of the environmental covariates were identified as effective predictors by the integrated model (Figure S1). The relationship of elevation, vegetation type, soil type, distance to waterways, and observational bias (distance to road) aligned with previous species distribution models (SDMs; Chang et al. 2022) and the known ecology of the species (e.g., Van Dyck 1993; Jackson 1998; Jackson et al. 2011). For a detailed discussion of the biological and ecological relevance of these predictors, see Chang et al. (2022).

Some discrepancies were observed. Precipitation seasonality, which was one of the key factors in previous SDMs (Chang et al. 2022), was not consistently significant in the integrated model. Notable differences were also found in the effects of fire frequency and temperature seasonality. Fire frequency was positively associated with abundance estimates in the integrated model; whereas it was not a significant predictor in previous SDMs. This is an interesting difference, as fire frequency is known to alter habitat suitability (Jackson et al. 2011). Temperature seasonality showed a positive correlation with abundance, which was an unexpected result based on previous SDMs (Chang et al. 2022) It is important to note that some environmental variables that may be important to the species, such as tree hollow availability, could not be included due to the lack of adequate spatial and field data (Chang et al. 2022).

### 4.3 Low Detectability and Integrated Model Performance

The estimated detectability of the Mahogany Glider ranged from 4.52% to 8.71% across our camera trapping sites. Low detectability is known to bias occupancy model estimates and reduce accuracy (e.g., MacKenzie et al. 2002; Field et al. 2005), with detectability below a suggested minimum threshold of 15% considered unsuitable for traditional occupancy models (O'Connell et al. 2006). An advantage of the integrated modelling approach is its ability to correct such biases by incorporating a species distribution model, thus improving accuracy at low detectability (Koshkina et al. 2017; Fidino 2021). However, it is worth noting that during the aggregation process, we retained only the camera with the highest detection counts at each site, which likely inflated the overall detectability. This, in turn, likely overestimated the total abundance of the species. This is one of the reasons that we lean towards the most conservative population estimate based on the 25 ha home range, which best represents the remaining habitat. Most suitable habitat is now concentrated in a few large patches, while many smaller patches appear to lack Mahogany Gliders (Chang et al. 2022).

Compared with the Royle–Nichols model, abundance heterogeneity in the integrated species distribution model is explained through the abundance intercept (latent intercept) and slope, which incorporate environmental covariates (Koshkina et al. 2017), rather than through site or time‐specific variation (Royle and Nichols 2003). Such variation in detectability can be implemented in the integrated model; for example, Koshkina et al. (2017) used wind strength and the time of day to implement varied detectability to estimate the abundance of Yellow‐bellied Gliders ( *Petaurus australis* ). Unfortunately, our surveys did not record covariates that may have contributed to detection variability. As a result, our model could only assume constant detectability across all camera locations and throughout the survey period. To improve future modeling efforts, surveys should aim to record potential sources of detectability variation, such as weather conditions, site characteristics and tree density.

Although a positive abundance–occupancy relationship is a common feature of ecological systems (He and Gaston 2000; Gaston et al. 2000), this relationship is not always linear. It can be influenced by factors such as population dynamics (Buckley and Freckleton 2010) or the spatial scale of the sampling unit (Storch et al. 2008). Both the integrated species distribution model and the Royle–Nichols model assume a nonlinear relationship between abundance and occupancy, where detection probability increases with abundance but eventually saturates (Royle and Nichols 2003; Koshkina et al. 2017; Fidino 2021).

### 4.4 Implications for Long‐Term Monitoring

Our findings highlight the critical role of long‐term monitoring in improving abundance modeling. As outlined above, a comparison of population density at Muller's Creek now versus 20 years ago (Jackson 2000a) suggests a substantial decline, with catch rates dropping from 7.5%–15% to just 2% (Chang et al. 2025). Standardized, long‐term data collection can estimate population trends through time and link these to changes in habitat quality (e.g., due to fire, intensity of cattle farming, invasive grasses, etc.), climate trends and rare events (e.g., cyclones). Sustained monitoring provides early warnings of population declines or evidence of recovery (e.g., Cassey et al. 2007; Rodhouse et al. 2019; Weldy et al. 2023; Harju and Cambrin 2023). For example, Cyclone Yasi in 2011 was believed to have had substantial negative impacts on Mahogany Glider populations (Starbridge 2012; Holloway 2013) but detailed monitoring data was lacking to assess population declines and subsequent recovery. Our study initiated a monitoring program, now led by local communities and conservation groups, using fixed arboreal camera traps at key sites. As these efforts continue and expand, abundance estimates for the Mahogany Glider will improve, enabling more effective conservation (McCarthy and Possingham 2007; Lindenmayer and Likens 2009).

### 4.5 Conclusions and Future Directions

The integrated model provides a baseline abundance estimate for this endangered species, especially in situations where traditional occupancy models struggle due to low detectability or large study areas (e.g., Linden et al. 2017; Koshkina et al. 2017). Despite limitations in accounting for population dynamics and variable home ranges, the model offers the best range‐wide population estimate to date for the Mahogany Glider. Based on the most conservative estimate from the integrated models, we suggest the current population sits at around 2800 individuals. To further refine these estimates, research should expand camera trapping efforts to additional areas, particularly poorly known areas of the distribution such as the base of the mountain ranges in the west and coastal areas in the east (Chang et al. 2022). Investigating other factors—such as the degree of habitat fragmentation, recent fire history, forest structure (tree size, age, tree hollows), floral species composition, flowering phenology—will be valuable in understanding variability in Mahogany Glider density and home range size. Combining these efforts with long‐term monitoring and advanced modeling techniques will enable more targeted and adaptive management of the species.

## Author Contributions

**Yiyin Chang:** conceptualization (equal), data curation (lead), formal analysis (equal), funding acquisition (equal), investigation (lead), methodology (equal), project administration (lead), validation (supporting), visualization (lead), writing – original draft (lead), writing – review and editing (lead). **Chieh Lin:** data curation (equal), formal analysis (equal), investigation (equal), methodology (equal), software (equal), validation (lead), writing – review and editing (supporting). **Conrad J. Hoskin:** conceptualization (equal), funding acquisition (lead), investigation (equal), methodology (equal), supervision (equal), validation (equal), writing – review and editing (equal).

## Ethics Statement

The research was conducted in accordance with Queensland Government Scientific Purposes Permits (protected areas: P‐PTUKI‐100021853; non‐protected areas: WA0025939) and James Cook University animal ethics (A2699).

## Conflicts of Interest

The authors declare no conflicts of interest.

## Supporting information


**Data S1:** Supporting Information.

## Data Availability

The R script used in this study is based on the GitHub repository of Mason Fidino (https://github.com/mfidino/integrated‐occupancy‐model) and is available on FigShare (DOI: https://doi.org/10.6084/m9.figshare.28448099.v3). Field data and additional datasets generated or analyzed during this study, including the species records, are available from the corresponding author upon reasonable request.

## References

[ece372037-bib-0001] Asari, Y. , C. N. Johnson , M. Parsons , and J. Larson . 2010. “Gap‐Crossing in Fragmented Habitats by Mahogany Gliders ( *Petaurus gracilis* ). Do They Cross Roads and Powerline Corridors?” Australian Mammalogy 32: 10. 10.1071/AM08017.

[ece372037-bib-0003] Bailey, L. L. , D. I. Mackenzie , and J. D. Nichols . 2014. “Advances and Applications of Occupancy Models.” Methods in Ecology and Evolution 5: 1269–1279. 10.1111/2041-210X.12100.

[ece372037-bib-0004] Bean, W. T. , R. Stafford , and J. S. Brashares . 2012. “The Effects of Small Sample Size and Sample Bias on Threshold Selection and Accuracy Assessment of Species Distribution Models.” Ecography 35: 250–258. 10.1111/J.1600-0587.2011.06545.

[ece372037-bib-0005] Blakeney, S. 2015. “Mahogany Glider/Core Habitat.” https://www.arcgis.com/home/item.html?id=568bf45585ae423f98fa2bf057b747eb&sublayer=0.

[ece372037-bib-0006] Bonar, S. A. , J. S. Fehmi , and N. Mercado‐Silva . 2011. “An Overview of Sampling Issues in Species Diversity and Abundance Surveys.” In Biological Diversity: Frontiers in Measurement and Assessment, 11–24. Oxford University Press.

[ece372037-bib-0007] Brooks, S. P. , and A. Gelman . 1998. “General Methods for Monitoring Convergence of Iterative Simulations.” Journal of Computational and Graphical Statistics 7: 434–455. 10.1080/10618600.1998.10474787.

[ece372037-bib-0008] Buckland, S. T. , E. A. Rexstad , T. A. Marques , and C. S. Oedekoven . 2015. Distance Sampling: Methods and Applications. Springer. 10.1007/978-3-319-19219-2.

[ece372037-bib-0009] Buckley, H. L. , and R. P. Freckleton . 2010. “Understanding the Role of Species Dynamics in Abundance‐Occupancy Relationships.” Journal of Ecology 98: 645–658. 10.1111/J.1365-2745.2010.01650.

[ece372037-bib-0010] Burbidge, A. , J. Woinarski , and P. Harrison . 2014. Action Plan for Australian Mammals 2012. CSIRO Publishing. 10.1071/9780643108745.

[ece372037-bib-0011] Burns, P. A. , C. McCall , K. C. Rowe , M. L. Parrott , and B. L. Phillips . 2019. “Accounting for Detectability and Abundance in Survey Design for a Declining Species.” Diversity and Distributions 25: 1655–1665. 10.1111/DDI.12966.

[ece372037-bib-0012] Cassey, P. , J. L. Lockwood , and K. H. Fenn . 2007. “Using Long‐Term Occupancy Information to Inform the Management of Cape Sable Seaside Sparrows in the Everglades.” Biological Conservation 139: 139–149. 10.1016/J.BIOCON.2007.06.010.

[ece372037-bib-0014] Chang, Y. , L. V. Bertola , and C. J. Hoskin . 2022. “Species Distribution Modelling of the Endangered Mahogany Glider (*Petaurus gracilis*) Reveals Key Areas for Targeted Survey and Conservation.” Austral Ecology 48: 289–312. 10.1111/aec.13266.

[ece372037-bib-0079] Chang, Y. , L. V. Bertola , K. R. Zenger , and C. J. Hoskin . 2025. “Conservation Genetics of Mahogany Gliders and Their Complex Evolutionary Relationship with Squirrel Gliders.” Conservation Genetics 26, no. 4: 731–750. 10.1007/s10592-025-01700-7.

[ece372037-bib-0016] Cunningham, R. B. , and D. B. Lindenmayer . 2005. “Modeling Count Data of Rare Species: Some Statistical Issues.” Ecology 86: 1135–1142. 10.1890/04-0589.

[ece372037-bib-0018] Dénes, F. V. , L. F. Silveira , and S. R. Beissinger . 2015. “Estimating Abundance of Unmarked Animal Populations: Accounting for Imperfect Detection and Other Sources of Zero Inflation.” Methods in Ecology and Evolution 6: 543–556. 10.1111/2041-210X.12333.

[ece372037-bib-0019] Denwood, M. J. 2016. “Runjags: An R Package Providing Interface Utilities, Model Templates, Parallel Computing Methods and Additional Distributions for MCMC Models in JAGS.” Journal of Statistical Software 71: i09. 10.18637/jss.v071.i09.

[ece372037-bib-0020] Dorazio, R. M. 2014. “Accounting for Imperfect Detection and Survey Bias in Statistical Analysis of Presence‐Only Data.” Global Ecology and Biogeography 23: 1472–1484. 10.1111/geb.12216.

[ece372037-bib-0022] Fidino, M. 2021. “A Gentle Introduction to an Integrated Occupancy Model That Combines Presence‐Only and Detection/Non‐Detection Data, and How to Fit it in JAGS.” https://github.com/dhope/integrated‐occupancy‐model.

[ece372037-bib-0082] Field, S. A. , A. J. Tyre , K. H. Thorn , P. J. O’Connor , and H. P. Possingham . 2005. “Improving the Efficiency of Wildlife Monitoring by Estimating Detectability: A Case Study of Foxes (*Vulpes vulpes*) on the Eyre Peninsula.” South Australia. Wildlife Research 32, no. 3: 253. 10.1071/wr05010.

[ece372037-bib-0084] Gaston, K. J. , T. M. Blackburn , J. J. D. Greenwood , R. D. Gregory , R. M. Quinn , and J. H. Lawton . 2000. “Abundance–Occupancy Relationships.” Journal of Applied Ecology 37, no. s1: 39–59. 10.1046/j.1365-2664.2000.00485.x.

[ece372037-bib-0025] Gerrodette, T. 1987. “A Power Analysis for Detecting Trends.” Ecology 68: 1364–1372. 10.2307/1939220.

[ece372037-bib-0081] Gilbert, N. A. , J. D. J. Clare , J. L. Stenglein , and B. Zuckerberg . 2020. “Abundance Estimation of Unmarked Animals Based on Camera‐Trap Data.” Conservation Biology 35, no. 1: 88–100. 10.1111/cobi.13517. 32297655

[ece372037-bib-0026] Gilbert, N. A. , J. D. J. Clare , J. L. Stenglein , and B. Zuckerberg . 2021. “Abundance Estimation of Unmarked Animals Based on Camera‐Trap Data.” Conservation Biology 35: 88–100. 10.1111/COBI.13517. 32297655

[ece372037-bib-0080] Gu, W. , and R. K. Swihart . 2004. “Absent or Undetected? Effects of Non‐Detection of Species Occurrence on Wildlife–Habitat Models.” Biological Conservation 116, no. 2: 195–203. 10.1016/s0006-3207(03)00190-3.

[ece372037-bib-0029] Harju, S. , and S. Cambrin . 2023. “Designing a Long‐Term Occupancy Monitoring Plan for a Cryptic Reptile.” Journal of Herpetology 57: 20–26. 10.1670/21-087.

[ece372037-bib-0083] He, F. , and K. J. Gaston . 2000. “Occupancy‐Abundance Relationships and Sampling Scales.” Ecography 23, no. 4: 503–511. 10.1111/j.1600-0587.2000.tb00306.x.

[ece372037-bib-0030] Holloway, I. 2013. “Effects of Cyclone Yasi on Vegetation Communities in the Tully/Mission Beach Area.”

[ece372037-bib-0031] Jackson, S. M. 1998. “Foraging Ecology, Behaviour and Management of the Mahogany Glider *Petaurus gracilis*.” http://researchonline.jcu.edu.au/17428/.

[ece372037-bib-0032] Jackson, S. M. 2000a. “Population Dynamics and Life History of the Mahogany Glider, *Petaurus gracilis*, and the Sugar Glider, *Petaurus breviceps*, in North Queensland.” Wildlife Research 27, no. 1: 21–37. 10.1071/WR98044.

[ece372037-bib-0033] Jackson, S. M. 2000b. “Home‐Range and Den Use of the Mahogany Glider, *Petaurus gracilis* .” Wildlife Research 27: 49–60. 10.1071/WR98046.

[ece372037-bib-0034] Jackson, S. M. , and A. Claridge . 1999. “Climatic Modelling of the Distribution of the Mahogany Glider (*Petaurus gracilis*), and the Squirrel Glider *(P. norfolcensis)* .” Australian Journal of Zoology 47: 47–57. 10.1071/ZO98044.

[ece372037-bib-0035] Jackson, S. M. , and J. Diggins . 2020. National Recovery Plan for the Mahogany Glider ( *Petaurus gracilis* ). DCCEEW Australian Government.

[ece372037-bib-0036] Jackson, S. M. , G. Morgan , J. E. Kemp , M. Maughan , and C. M. Stafford . 2011. “An Accurate Assessment of Habitat Loss and Current Threats to the Mahogany Glider ( *Petaurus gracilis* ).” Australian Mammalogy 33: 82–92.

[ece372037-bib-0037] Jackson, S. M. , M. Parsons , M. Baseler , and D. Stanton . 2019. “Landscape Management of the Mahogany Glider *(Petaurus gracilis)* Across Its Distribution: Subpopulations and Corridor Priorities.” Australian Mammalogy 42: 152–159. 10.1071/AM19010.

[ece372037-bib-0038] Jeliazkov, A. , Y. Gavish , C. J. Marsh , et al. 2022. “Sampling and Modelling Rare Species: Conceptual Guidelines for the Neglected Majority.” Global Change Biology 28: 3754–3777. 10.1111/GCB.16114. 35098624

[ece372037-bib-0040] Kays, R. , B. S. Arbogast , M. Baker‐Whatton , et al. 2020. “An Empirical Evaluation of Camera Trap Study Design: How Many, How Long and When?” Methods in Ecology and Evolution 11: 700–713. 10.1111/2041-210X.13370.

[ece372037-bib-0041] Koshkina, V. , Y. Wang , A. Gordon , R. M. Dorazio , M. White , and L. Stone . 2017. “Integrated Species Distribution Models: Combining Presence‐Background Data and Site‐Occupancy Data With Imperfect Detection.” Methods in Ecology and Evolution 8: 420–430. 10.1111/2041-210X.12738.

[ece372037-bib-0042] Kruschke, J. K. 2014. Doing Bayesian Data Analysis: A Tutorial With R, JAGS, and Stan, Second Edition. Academic Press. 10.1016/B978-0-12-405888-0.09999-2.

[ece372037-bib-0044] Linden, D. W. , A. K. Fuller , J. A. Royle , and M. P. Hare . 2017. “Examining the Occupancy–Density Relationship for a Low‐Density Carnivore.” Journal of Applied Ecology 54: 2043–2052. 10.1111/1365-2664.12883.

[ece372037-bib-0045] Lindenmayer, D. B. , and G. E. Likens . 2009. “Adaptive Monitoring: A New Paradigm for Long‐Term Research and Monitoring.” Trends in Ecology & Evolution 24: 482–486. 10.1016/j.tree.2009.03.005. 19409648

[ece372037-bib-0046] Link, W. A. , and J. R. Sauer . 1997. “Estimation of Population Trajectories From Count Data.” Biometrics 53: 488–497. 10.2307/2533952.

[ece372037-bib-0047] MacKenzie, D. I. , and J. D. Nichols . 2004. “Occupancy as a Surrogate for Abundance Estimation.” Animal Biodiversity and Conservation 27: 461–467. https://raco.cat/index.php/ABC/article/view/57325.

[ece372037-bib-0048] MacKenzie, D. I. , J. D. Nichols , G. B. Lachman , S. Droege , J. A. Royle , and C. A. Langtimm . 2002. “Estimating Site Occupancy Rates When Detection Probabilities Are Less Than One.” Ecology 83: 2248. 10.2307/3072056.

[ece372037-bib-0050] Mäkinen, J. , C. Merow , and W. Jetz . 2024. “Integrated Species Distribution Models to Account for Sampling Biases and Improve Range‐Wide Occurrence Predictions.” Global Ecology and Biogeography 33: 356–370. 10.1111/GEB.13792.

[ece372037-bib-0051] McCarthy, M. A. , and H. P. Possingham . 2007. “Active Adaptive Management for Conservation.” Conservation Biology 21: 956–963. 10.1111/j.1523-1739.2007.00677.x. 17650246

[ece372037-bib-0054] Nakashima, Y. 2020. “Potentiality and Limitations of N‐Mixture and Royle‐Nichols Models to Estimate Animal Abundance Based on Non‐Instantaneous Point Surveys.” Population Ecology 62: 151–157. 10.1002/1438-390X.12028.

[ece372037-bib-0056] O'Connell, A. F. , N. W. Talancy , L. L. Bailey , J. R. Sauer , R. Cook , and A. T. Gilbert . 2006. “Estimating Site Occupancy and Detection Probability Parameters for Meso‐ and Large Mammals in a Coastal Ecosystem.” Journal of Wildlife Management 70, no. 6: 1625–1633. 10.2193/0022-541X(2006)70.

[ece372037-bib-0059] Plummer, M. 2003. “JAGS: A Program for Analysis of Bayesian Graphical Models Using Gibbs Sampling JAGS: Just Another Gibbs Sampler.” r‐project.org.

[ece372037-bib-0060] R Development Core Team . 2022. R: A Language and Environment for Statistical Computing.

[ece372037-bib-0061] Rodhouse, T. J. , R. M. Rodriguez , K. M. Banner , P. C. Ormsbee , J. Barnett , and K. M. Irvine . 2019. “Evidence of Region‐Wide Bat Population Decline From Long‐Term Monitoring and Bayesian Occupancy Models With Empirically Informed Priors.” Ecology and Evolution 9: 11078–11088. 10.1002/ECE3.5612. 31641456 PMC6802066

[ece372037-bib-0062] Rowcliffe, J. M. , J. Field , S. T. Turvey , and C. Carbone . 2008. “Estimating Animal Density Using Camera Traps Without the Need for Individual Recognition.” Journal of Applied Ecology 45: 1228–1236. 10.1111/j.1365-2664.2008.01473.x.

[ece372037-bib-0063] Royle, J. A. 2004. “N‐Mixture Models for Estimating Population Size From Spatially Replicated Counts.” Biometrics 60: 108–115. 10.1111/J.0006-341X.2004.00142.X. 15032780

[ece372037-bib-0064] Royle, J. A. , and J. D. Nichols . 2003. “Estimating Abundance From Repeated Presence–Absence Data or Point Counts.” Ecology 84: 777–790. 10.1890/0012-9658(2003)084[0777:EAFRPA]2.0.CO;2.

[ece372037-bib-0065] Royle, J. A. , J. D. Nichols , and M. Kéry . 2005. “Modelling Occurrence and Abundance of Species When Detection Is Imperfect.” Oikos 110: 353–359. 10.1111/j.0030-1299.2005.13534.x.

[ece372037-bib-0066] Schank, C. J. , M. V. Cove , M. J. Kelly , et al. 2017. “Using a Novel Model Approach to Assess the Distribution and Conservation Status of the Endangered Baird's Tapir.” 23: 1459–1465. 10.1111/ddi.12631.

[ece372037-bib-0067] Schank, C. J. , M. V. Cove , M. J. Kelly , et al. 2019. “A Sensitivity Analysis of the Application of Integrated Species Distribution Models to Mobile Species: A Case Study With the Endangered Baird's Tapir.” Environmental Conservation 46: 184–192. 10.1017/S0376892919000055.

[ece372037-bib-0069] Starbridge, S. 2012. “Getting Through a Rough Patch: Cyclone Yasi and Mahogany Glider Habitat.” Wildlife Australia 49: 18–21.

[ece372037-bib-0071] Steenweg, R. , M. Hebblewhite , J. Whittington , P. Lukacs , and K. McKelvey . 2018. “Sampling Scales Define Occupancy and Underlying Occupancy–Abundance Relationships in Animals.” Ecology 99: 172–183. 10.1002/ecy.2054. 29065232

[ece372037-bib-0085] Storch, D. , A. L. Šizling , J. Reif , J. Polechová , E. Šizlingová , and K. J. Gaston . 2008. “The Quest for a Null Model for Macroecological Patterns: Geometry of Species Distributions at Multiple Spatial Scales.” Ecology Letters 11, no. 8: 771–784. 10.1111/j.1461-0248.2008.01206.x. 18638301

[ece372037-bib-0072] Threatened Species Scientific Committee . 2023. “EPBC Act List of Threatened Fauna.” https://www.environment.gov.au/cgi‐bin/sprat/public/publicthreatenedlist.pl#mammals_extinct.

[ece372037-bib-0073] Twining, J. P. , A. K. Fuller , C. C. Sun , et al. 2024. “Integrating Presence‐Only and Detection/Non‐Detection Data to Estimate Distributions and Expected Abundance of Difficult‐To‐Monitor Species on a Landscape‐Scale.” Journal of Applied Ecology 61: 1441–1459. 10.1111/1365-2664.14633.

[ece372037-bib-0074] Van Dyck, S. 1993. “The Taxonomy and Distribution of *Petaurus gracilis* (Marsupialia: Petauridae), With Notes on Its Ecology and Conservation Status.” Memoirs of the Queensland Museum 33: 122.

[ece372037-bib-0075] Weldy, M. J. , D. B. Lesmeister , C. B. Yackulic , C. L. Appel , C. McCafferty , and J. David Wiens . 2023. “Long‐Term Monitoring in Transition: Resolving Spatial Mismatch and Integrating Multistate Occupancy Data.” Ecological Indicators 146: 109815. 10.1016/J.ECOLIND.2022.109815.

[ece372037-bib-0076] Welsh, A. H. , R. B. Cunningham , C. F. Donnelly , and D. B. Lindenmayer . 1996. “Modelling the Abundance of Rare Species: Statistical Models for Counts With Extra Zeros.” Ecological Modelling 88: 297–308. 10.1016/0304-3800(95)00113-1.

[ece372037-bib-0077] White, E. R. 2019. “Minimum Time Required to Detect Population Trends: The Need for Long‐Term Monitoring Programs.” Bioscience 69, no. 1: 40–46. 10.1093/BIOSCI/BIY144.

[ece372037-bib-0078] White, G. C. , and K. P. Burnham . 1999. “Program MARK: Survival Estimation From Populations of Marked Animals.” Bird Study 46: S120–S139. 10.1080/00063659909477239.

