# Supplementary material for: Estimating the Abundance of an Endangered Arboreal Marsupial Using Camera Traps and an Integrated Species Distribution Model
Source: Ecol Evol. 2025 Sep 1;15(9):e72037. doi: 10.1002/ece3.72037 (PMC12401547; doi:10.1002/ece3.72037)

# Appendix

### Table S1

The table shows the predictors employed in the integrated species distribution model. The predictors were selected from the subset of variables identified by the latest species distribution model of the species (Chang *et al.*2022). Categorical variables were transformed into binary values, with 1 and 0 denoting the effective and non-effective category, respectively. The values of each predictor variable were appropriately scaled and centred prior to the modelling process.

| Predictor variables | Year coverage | Units | Source |
| --- | --- | --- | --- |
| Bio_04: Temperature Seasonality | 1996–2015 | °C | Storlie *et al.*2013 |
| Bio_06: Min Temperature of Coldest Month | 1996–2015 | °C | Storlie *et al.*2013 |
| Bio_15: Precipitation Seasonality | 1996–2015 | mm | Storlie *et al.*2013 |
| Elevation | 2007 | meters | Department of Natural Resources, Mines and Energy, 2005 |
| Slope | 2007 | degrees |  |
| Distance to major water ways | 2016 | meters | Queensland Government Department of Agriculture and Fisheries, 2016 |
| Fire Frequency AVHRR | 1997–2011 | 0–7 burnt times | Maier and Russell-Smith, 2012 |
| Soil: Hydrosol | 2011 | 1, 0 | Australian Collaborative Land Evaluation Program, 2014 |
| Vegetation: 9 and 15^†^ | pre-1750, 2006 | 1, 0 | Department of Environment and Science, 2012 |
| †Eucalyptus woodlands with a tussock grass understory, Melaleuca open forests and woodlands | | | |

### Table S2

Coefficient estimates for the integrated model at aggregation factors of 3 (9 ha), 4 (16 ha), and 5 (25 ha). Coefficients are presented as medians with their 95% credible intervals, along with means and standard deviations (SD). The effective sample size is denoted as SSeff, and the Gelman-Rubin diagnostic is reported as PSRF. Note that the coefficient estimates below are one of 1,000 sets of model coefficients from the posterior distribution.

|  | Lower95 | Median | Upper95 | Mean | SD | SSeff | PSRF |
| --- | --- | --- | --- | --- | --- | --- | --- |
| Aggregation factor = 3 | | | | | | | |
| SDM Intercept | -4.28 | -3.17 | -2.21 | -3.2 | 0.53 | 4030 | 1 |
| Temperature seasonality | 0.32 | 0.63 | 0.93 | 0.63 | 0.15 | 10373 | 1 |
| Minimal temperature of the coldest month | -0.06 | 0.38 | 0.85 | 0.38 | 0.23 | 9307 | 1 |
| Precipitation seasonality | 0.08 | 0.24 | 0.41 | 0.24 | 0.09 | 12598 | 1 |
| Elevation | -4.43 | -3.37 | -2.37 | -3.4 | 0.53 | 3666 | 1 |
| Slope | -0.1 | 0.23 | 0.56 | 0.23 | 0.17 | 6088 | 1 |
| Fire Frequency | 0.09 | 0.34 | 0.57 | 0.34 | 0.12 | 18469 | 1 |
| Soil (Hydrosol) | -0.02 | 0.08 | 0.18 | 0.08 | 0.05 | 20204 | 1 |
| Vegetation type | 0.28 | 0.4 | 0.52 | 0.4 | 0.06 | 18367 | 1 |
| Distance to waterways | 0.05 | 0.19 | 0.32 | 0.19 | 0.07 | 18616 | 1 |
| PO intercept | -6.86 | -4.02 | -1.94 | -4.22 | 1.32 | 19729 | 1 |
| Observation bias | -2.68 | -2.05 | -1.45 | -2.06 | 0.31 | 20234 | 1 |
| Occupancy intercept | -2.6 | -2.35 | -2.12 | -2.36 | 0.12 | 20443 | 1 |
| Occupancy estimate | 3253 | 5254 | 7390 | 5321.34 | 1073.7 | 14959 | 1 |
| Abundance estimate | 3527 | 5879 | 8658 | 5981.18 | 1339.9 | 15363 | 1 |
| Aggregation factor = 4 | | | | | | | |
| Intercept | -4.61 | -3.38 | -2.36 | -3.41 | 0.57 | 3792 | 1 |
| Temperature seasonality | 0.32 | 0.64 | 0.95 | 0.64 | 0.16 | 10601 | 1 |
| Minimal temperature of the coldest month | -0.16 | 0.31 | 0.77 | 0.31 | 0.24 | 9712 | 1 |
| Precipitation seasonality | 0.01 | 0.19 | 0.36 | 0.19 | 0.09 | 11630 | 1 |
| Elevation | -4.66 | -3.45 | -2.43 | -3.49 | 0.57 | 3286 | 1 |
| Slope | -0.03 | 0.31 | 0.65 | 0.31 | 0.17 | 5171 | 1 |
| Fire Frequency | -0.02 | 0.26 | 0.51 | 0.26 | 0.14 | 19733 | 1 |
| Soil (Hydrosol) | -0.01 | 0.1 | 0.21 | 0.1 | 0.05 | 20000 | 1 |
| Vegetation type | 0.26 | 0.39 | 0.51 | 0.39 | 0.07 | 18245 | 1 |
| Distance to waterways | 0.03 | 0.17 | 0.31 | 0.17 | 0.07 | 18476 | 1 |
| PO intercept | -6.76 | -3.95 | -1.91 | -4.16 | 1.29 | 19259 | 1 |
| Observation bias | -2.93 | -2.24 | -1.59 | -2.25 | 0.35 | 19654 | 1 |
| Occupancy intercept | -2.7 | -2.44 | -2.19 | -2.44 | 0.13 | 20467 | 1 |
| Occupancy estimate | 2570 | 4161 | 5943 | 4217.66 | 874.74 | 15666 | 1 |
| Abundance estimate | 2757 | 4804 | 7213 | 4906.21 | 1164.45 | 16272 | 1 |
| Aggregation factor = 5 | | | | | | | |
| Intercept | -5.18 | -3.91 | -2.81 | -3.94 | 0.6 | 2248 | 1 |
| Temperature seasonality | 0.35 | 0.67 | 1.01 | 0.67 | 0.17 | 5043 | 1 |
| Minimal temperature of the coldest month | -0.23 | 0.26 | 0.75 | 0.26 | 0.25 | 4156 | 1 |
| Precipitation seasonality | -0.08 | 0.1 | 0.28 | 0.1 | 0.09 | 6904 | 1 |
| Elevation | -4.62 | -3.45 | -2.42 | -3.48 | 0.56 | 1939 | 1 |
| Slope | -0.06 | 0.27 | 0.63 | 0.27 | 0.18 | 3239 | 1 |
| Fire Frequency | 0.04 | 0.31 | 0.56 | 0.3 | 0.13 | 13670 | 1 |
| Soil (Hydrosol) | 0.04 | 0.15 | 0.26 | 0.15 | 0.06 | 14601 | 1 |
| Vegetation type | 0.3 | 0.44 | 0.57 | 0.44 | 0.07 | 12525 | 1 |
| Distance to waterways | 0.01 | 0.16 | 0.31 | 0.16 | 0.08 | 13396 | 1 |
| PO intercept | -6.48 | -3.55 | -1.58 | -3.75 | 1.3 | 13684 | 1 |
| Observation bias | -2.76 | -2.04 | -1.4 | -2.06 | 0.35 | 15888 | 1 |
| Occupancy intercept | -3.6 | -3.05 | -2.59 | -3.07 | 0.26 | 8519 | 1 |
| Occupancy estimate | 1197 | 2424 | 3948 | 2511.68 | 739.68 | 7245 | 1 |
| Abundance estimate | 1253 | 2795 | 4952 | 2945.53 | 1024.44 | 7327 | 1 |

### Fig. S1

### Caterpillar plot showing the coefficient estimates of the integrated model when the aggregation factor is equal to 4 (16-hectare home range). These coefficients represent environmental covariates that affect the probability of occupancy across aggregated cells. Most covariates were effective predictors, as indicated by credible intervals that did not overlap zero. The coefficients remained relatively consistent across the aggregation factors (the three home range scenarios).

###
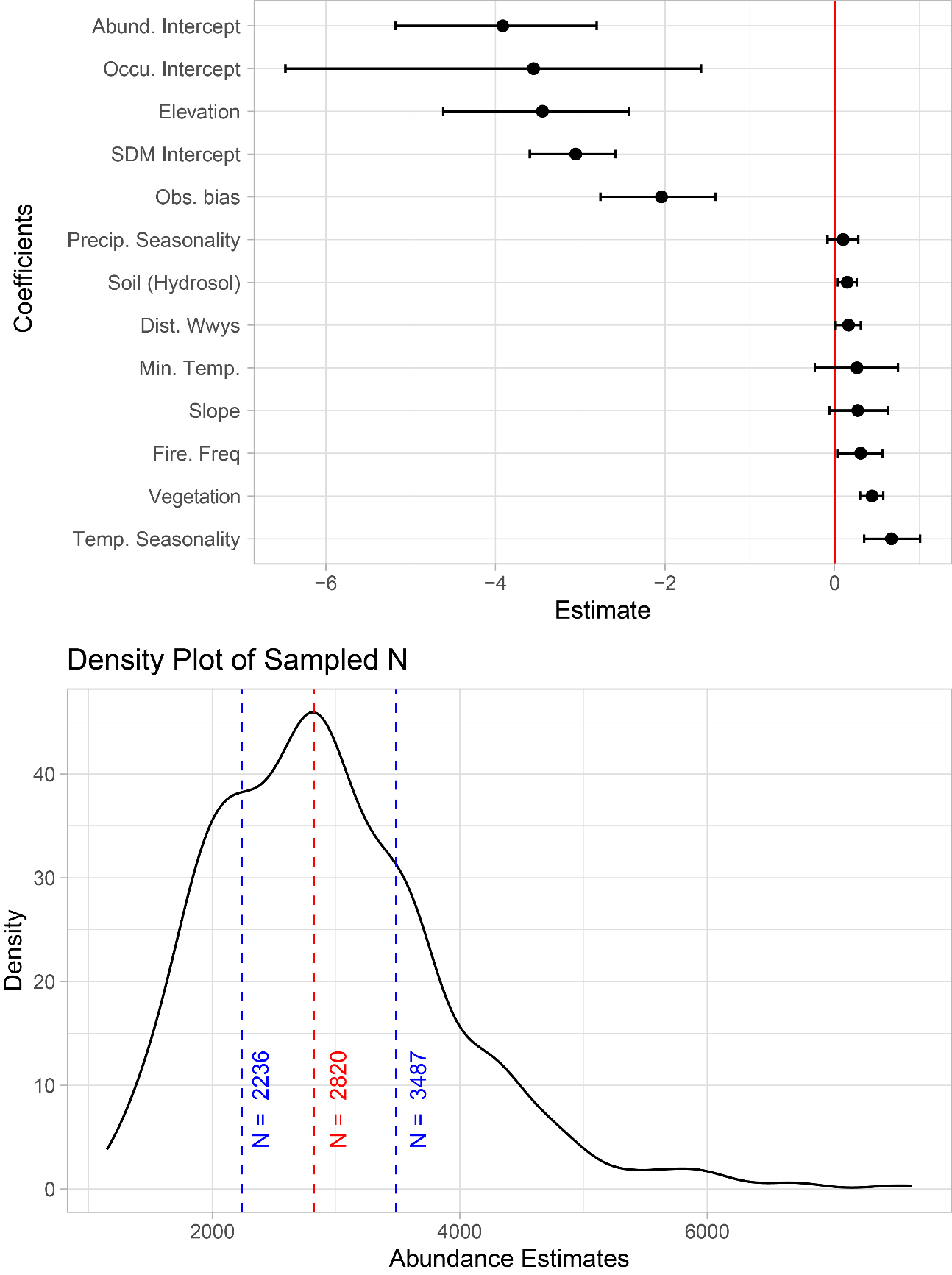


### Fig. S2


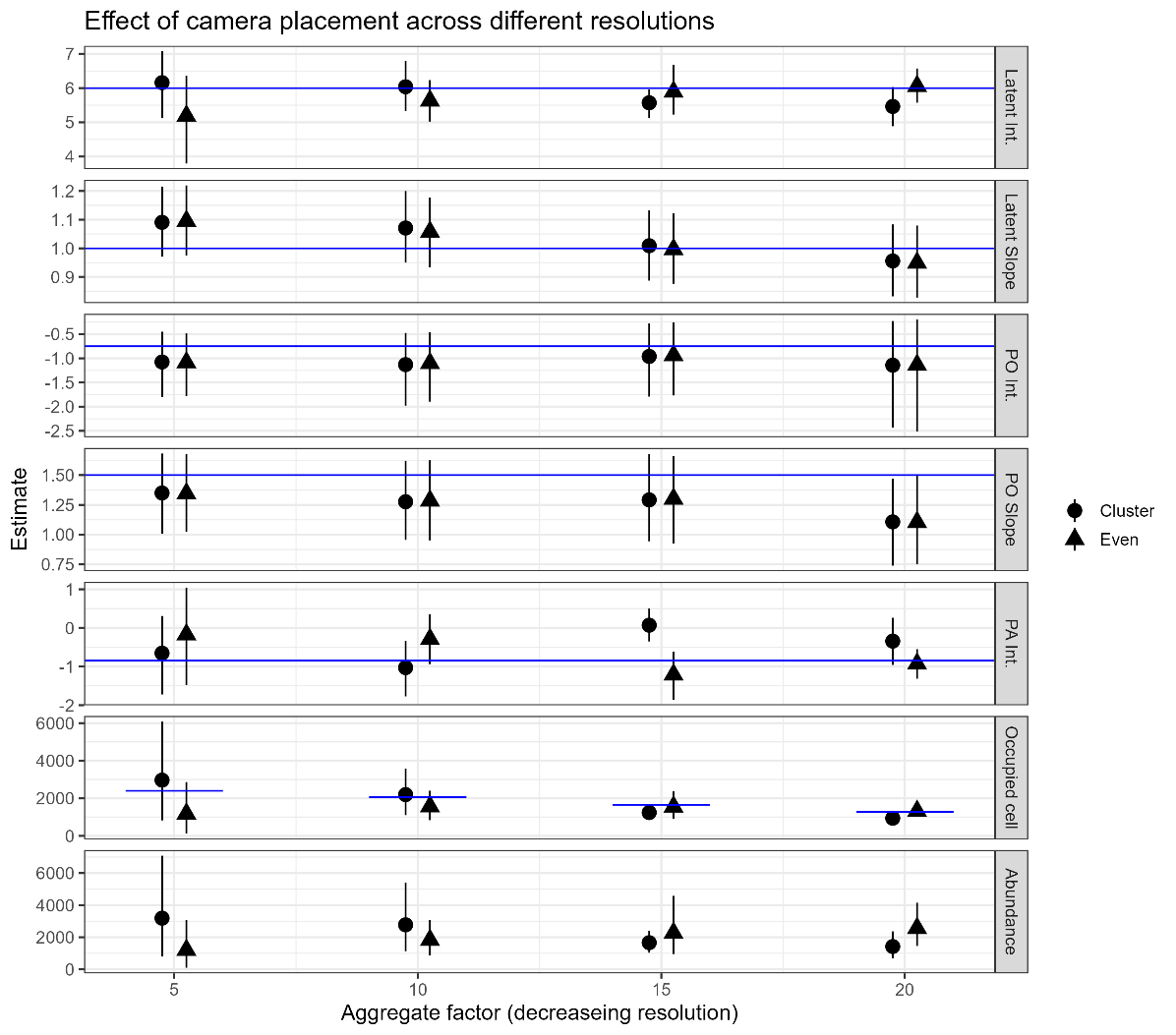
The plot demonstrates the effect of camera placement across different resolutions (aggregation factors). Simulated data were used to test clustered versus evenly placed cameras. The blue lines represent the assigned coefficients, while the points and 95% credible intervals represent estimated coefficients. The circles and triangles indicate the medians of clustered and evenly placed camera placements, respectively. The plot illustrates whether the estimates capture the assigned true values, which indicate reliability. The assigned coefficients are Abundance Intercept (Latent Int.), Environmental Covariate Coefficient (Latent Slope), SDM Intercept (PO Int.), Observation Bias (PO slope), Occupancy Intercept (PA Int.). The derived values from the assigned coefficients are Occupied cells that subsequently estimates the Abundance.

### Fig. S3


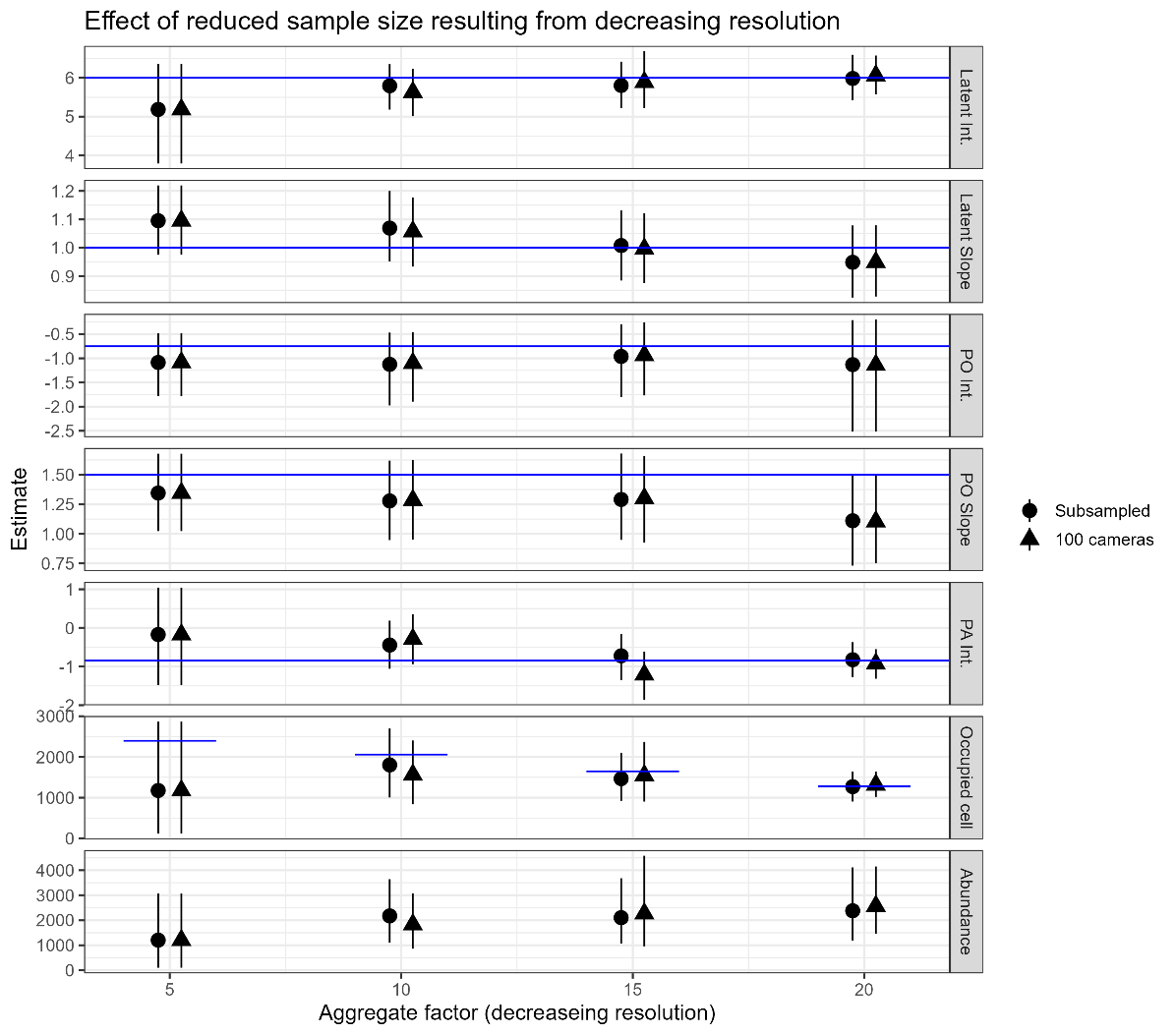
The plot demonstrates the effect of reduced sample size resulted from subsampling due to the increasing aggregation factors (decreasing resolutions). Simulated data were used to test subsampled cameras versus original 100 cameras. All cameras were evenly distributed before subsampling. As spatial resolution decrease, cameras were randomly removed from the raster to mimic the pattern of observed data. The blue lines represent the assigned coefficients, while the points and 95% credible intervals represent estimated coefficients. The circles and triangles indicate the medians of subsampled and original camera numbers, respectively. The plot illustrates whether the estimates capture the true values, which indicate reliability. The assigned coefficients are Abundance Intercept (Latent Int.), Environmental Covariate Coefficient (Latent Slope), SDM Intercept (PO Int.), Observation Bias (PO slope), Occupancy Intercept (PA Int.). The derived values from the assigned coefficients are Occupied cells that subsequently estimates the Abundance.

### Fig. S4

The plot demonstrates the effect of different aggregation methods (max, mean, sum) across various resolutions (aggregation factors) using simulated data. All cameras were evenly distributed before the aggregation. The blue lines represent the assigned coefficients, while the points and 95% credible intervals represent estimated coefficients. The circles and triangles indicate the medians of subsampled and original camera numbers, respectively. The plot illustrates whether the estimates capture the true values, which indicate reliability. The assigned coefficients are Abundance Intercept (Latent Int.), Environmental Covariate Coefficient (Latent Slope), SDM Intercept (PO Int.), Observation Bias (PO slope), Occupancy Intercept (PA Int.). The derived values from the assigned coefficients are Occupied cells that subsequently estimates the Abundance.


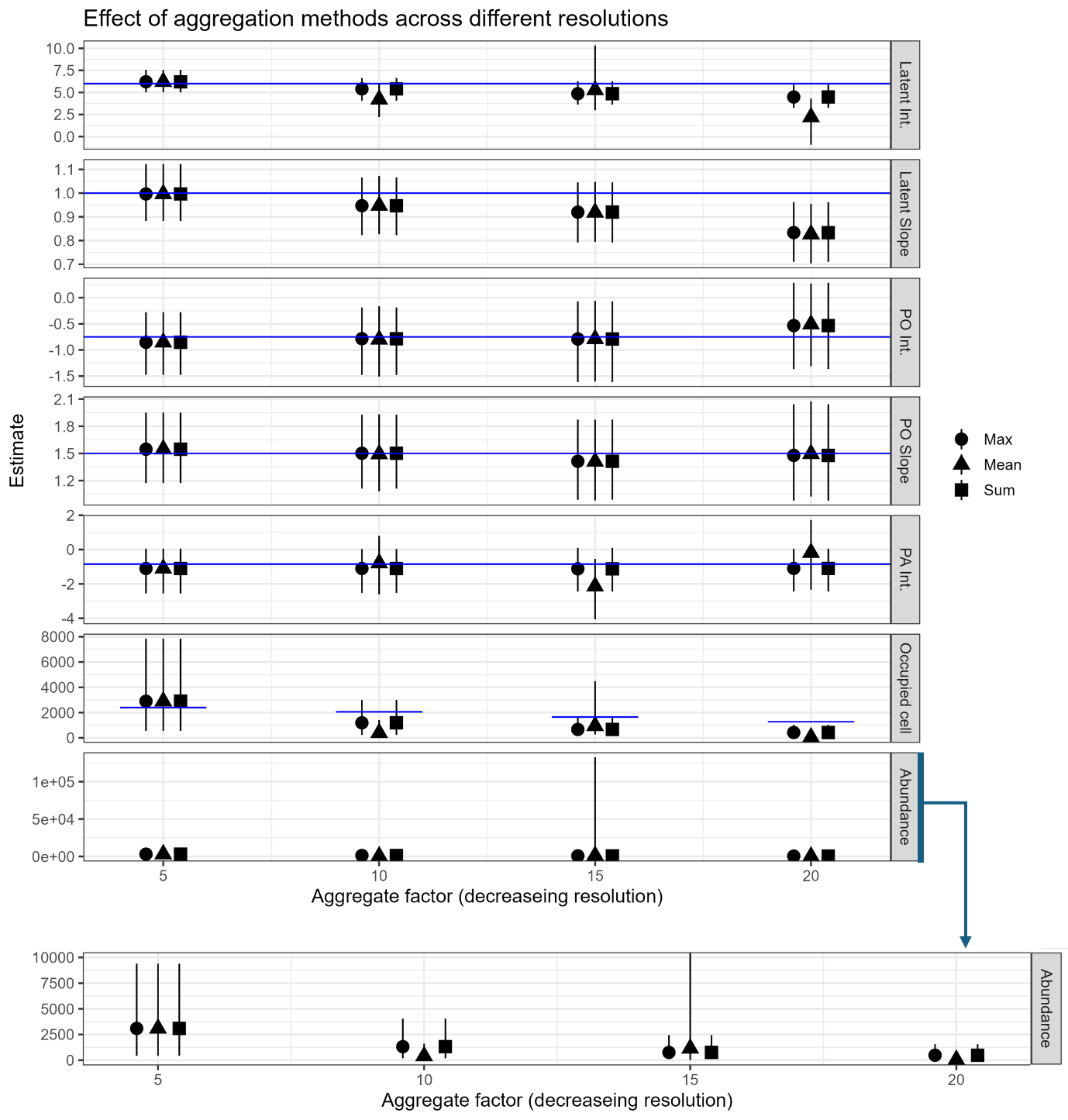

Supplement: Supplementary file 1 — Data S1: Supporting Information. [file ECE3-15-e72037-s001.docx]
